# Impact of Image Filters and Observations Parameters in CBCT for Identification of Mandibular Osteolytic Lesions

**DOI:** 10.1155/2012/239306

**Published:** 2012-08-22

**Authors:** Bruna Moraes Monteiro, Denys Silveira Nobrega Filho, Patrícia de Medeiros Loureiro Lopes, Marcelo Augusto Oliveira de Sales

**Affiliations:** ^1^Oral Diagnosis, Federal University of Paraiba, João Pessoa, PB, Brazil; ^2^University of São Paulo, São Paulo, SP, Brazil; ^3^Department of Clinical and Social Dentistry, Federal University of Paraiba, João Pessoa, PB, Brazil; ^4^Department of Clinical and Social Dentistry, Cidade Universitária, 58051-900 João Pessoa, PB, Brazil

## Abstract

The aim of this study was to analyze the influence of filters (algorithms) to improve the image of Cone Beam Computed Tomography (CBCT) in diagnosis of osteolytic lesions of the mandible, in order to establish the protocols for viewing images more suitable for CBCT diagnostics. 15 dry mandibles in which perforations were performed, simulating lesions, were submitted to CBCT examination. Two examiners analyzed the images, using filters to improve image Hard, Normal, and Very Sharp, contained in the iCAT Vision software, and protocols for assessment: axial; sagittal and coronal; and axial, sagittal and coronal planes simultaneously (MPR), on two occasions. The sensitivity and specificity (validity) of the cone beam computed tomography (CBCT) have been demonstrated as the values achieved were above 75% for sensitivity and above 85% for specificity, reaching around 95.5% of sensitivity and 99% of specificity when we used the appropriate observation protocol. It was concluded that the use of filters (algorithms) to improve the CBCT image influences the diagnosis, due to the fact that all measured values were correspondingly higher when it was used the filter Very Sharp, which justifies its use for clinical activities, followed by Hard and Normal filters, in order of decreasing values.

## 1. Introduction

According to the parameters published by the American Academy of Oral and Maxillofacial Radiology (AAOMR), criteria should be used by dentists who are responsible for the clinical management of patients with anormalities in the oral and maxillofacial region [1].

Although the combination of conventional projections and panoramic radiographs is appropriate in a large number of clinical situations, radiographic assessment can often be facilitated by multiplanar image viewing, including the computed tomography images [2, 3].

Furthermore, technological evolvement results in improvement of diagnosis of bone lesions. In this regard, filters of refining the image can be used to further enhance CBCT images, producing better diagnosis of osteolytic lesions of the jaws. The evaluation of the presence of bone destructions is fundamental, since it directs the prognosis, planning, and conducting of the therapeutic process.

Following these statements, the purpose of this study was to analyze the impact of image filters and observations parameters in diagnostic enhancement of CBCT image, in order to establish protocols for CBCT interpretation, regarding the scope of mandibular osteolytic lesions diagnosis.

## 2. Methodology

The sample consisted by CBCT scans of 15 dry mandibles, in which lesions were produced involving only cortical or cortical and medullary, using a high-speed hand piece with dental carbide burs (sizes 1, 3, and 6 KG Sorensen, Cotia, SP, Brazil). The lesions were located in the buccal or lingual cortex of the mandibular body, and they presented different dimensions, shapes, and loci numbers, depending on the size of the used burr, in order to simulate uni- and multilocular lesions. In order to simulate the attenuation caused by soft tissue in an *in vivo* situation, the specimens were installed in a plastic container containing about 1 liter of water, so as to be totally submerged. Subsequently, the tomographic acquisition was performed using a cone beam device iCAT cone beam 3D dental Imaging System (Imaging Sciences International, Hatfield, PA, USA), with the following acquisition parameters: 0,25 mm of voxel size and 40 seconds of time of acquisition.

After acquisition of RAW data, the images were stored in natively universal DICOM format to avoid data loss and transferred to workstation located in an environment adjacent to console. Original volumes of CBCT scans were examined by two examiners, radiologists with previous experience in reports of CT scans, independently, who analyzed the images in the iCAT Vision software (version 1.6.20), without prior knowledge of any information on the simulated lesions, and the interpretation followed the principle of randomization of the images, on a personal computer, according to the established protocols of observation, on two occasions with an interval of seven consecutive days between assessments. The evaluation of artificial bone perforations followed the protocol of interpretation and was always the same for all images taken by the same examiner.

All assessments were conducted individually and sequenced (a filter to improve the image at a time) for each examiner and images in question, under controlled light and environment free of external stimuli.

The sequence analysis of the images complied with the following protocols:

1. evaluation of images obtained by CBCT acquisition on axial images with the use of following filters: hard, normal, and very sharp, through the program iCAT Vision in an independent workstation;
2. evaluation of images obtained by CBCT acquisition on coronal and sagittal protocols with the use of the same sequence of image filters;
3. evaluation of images obtained by CBCT acquisition on axial, coronal, and sagittal protocols simultaneously (MPR) with the use of the same sequence of image filters.

During the study of the images, only the studied image plane was displayed on the computer monitor, remaining the other pictures hidden using tools available in the program, in order to avoid interference in the assertion of presence or absence of mandibular lesion. Then, data were recorded properly for subsequent statistical analysis.

The statistical evaluation was performed by using validity test (sensitivity and specificity), positive predictive value (PPV), negative predictive value (NPV), and accuracy [4], to establish the validity of CBCT protocols in relation to the gold standard (macerated jaws), and in relation to graphic programs and methods of visualization used. In all evaluated itemswas considered the range of 95% (*P* < 0.5).

In order to check the reproducibility of the method above, testing intra- and interobserver agreement, were obtained Kappa scores including obtaining an interval of 95.0% of confidence for the population Kappa.

The significance level used in the decision of the statistical test was 5%. Data were entered via Excel 2007 spreadsheet, and the software used to obtain statistical calculations was SPSS version 17.0 for windows (Statistical Package for Social Sciences, Chicago, IL, USA) [5].

This work was submitted to the Ethics Committee of FOUSP under number 149/03, protocol 151/03.

## 3. Results

### 3.1. Study of Sensitivity (S), Specificity (Sp), Positive Predictive Value (PPV), Negative Predictive Value (NPV), and Accuracy (A) of the Methods in Relation to the Gold Standard

Tables 1 to 4 show the results of measures: sensitivity (S), specificity (Sp), positive predictive value (PPV), negative predictive value (NPV), and accuracy (A) for the evaluation criterion for the presence or absence of simulated lesions in the total group, according to the type of image enhancement filter used, according to the protocol type used, and according to the jaw area searched.

### 3.2. Study of the Degree of Agreement

Tables 5 to 7 show the results of the agreement of the examiners with the gold standard (intra- and interobserver agreement) in relation to the loci numbers.

## 4. Discussion

The cone beam computerized tomography (CBCT) was a big step in the improvement of CT examinations in dentistry, because the proposed improvements added quality to the image, facilitating diagnosis, and also increase the study, the facility, and the interest of dentists [1, 6].

 The use of cone beam computed tomography as a tool to perform morphologic and morphometric studies of the mandible is due to the advantages of this type of scan features, such as the observation of an extensive region of the mandibular arch, including areas and anatomical structures adjacent to the mandibular canal, allowing a precise analysis of the tract and its relationship with anatomical reference points [7, 8].

The use of reduced thicknesses cut and reduced sizes of voxels in CBCT allows greater sensitivity and specificity [9–11].

Our results found, in general, sensitivity above 75% and specificity above 85% (Tables 1 to 4). According to the observation protocol used, these values reach up to 95.5% of sensitivity and 99% of specificity (Tables 3 and 4). These findings demonstrate the influence of observation protocols on the interpretation of exams. From the data compiled, we can infer the high sensitivity and specificity of cone beam computed tomography, which corroborates the data obtained by Schulze et al., in 2006 [12].

The use of more advanced algorithms and the greatest number of visualization tools have an important role in determining the number of bone lesions in the jaws, as well as improving the process of diagnosis and treatment [13–17], thus justifying the use of independent workstations and different protocols observation.

**Figure 1:**
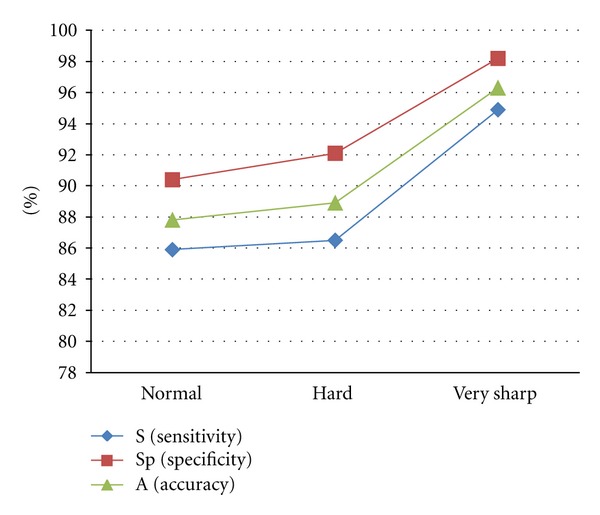
Evaluation of the sensitivity (S), specificity (Sp), and accuracy (A), according to the type of filter, in the study of the presence or absence of simulated lesions.

Suomalainen et al., in 2008 [18], said that the use of postprocessing filter in CBCT scans allowed better visualization of bone structures. These filters can be applied to soft or enhance the final image on the monitor screen. In our study, by the results in Table 2, we can see that all values were correspondingly higher when the filter very sharp was used and were less high when the filter normal was used in the study of the presence or absence of osteolytic simulated lesions (Figure 1). The sensitivity values ranged from 85,9% to 94.9%, the specific values ranged from 90.4% to 98.2%, and PPV ranged from 92.4% to 98.7%. The NPV value was 82.4% (Normal filter), and between the filters Hard (92.1%) and very sharp (93.3%) was approximated. The results of the accuracy ranged from 87.8% to 96.3%. From this table, auxiliary calculations show that the largest percentage difference between the filter types occurred in the NPV, 10.9% higher when the filter very sharp was used than the value for normal filter (93.3% × 82.4%).

According to the manual of use of the iCAT Vision software, patterns of use of filters to improve the images to be followed are the following:

1. initial screen display: hard filter for all images,
2. screen for planning implants: hard filter for all images,
3. screen for ATM viewing: hard filter for the 3 first images and normal filter to Condyle images in preview mode Ortho,
4. MPR screen: normal filter for all images,
5. Cephalometric screen (Ortho view mode): very sharp image filter for left and right top images and hard filter for all others.

According to the results obtained in our research, Normal filter was inferior for the visualization and detection of simulated osteolytic lesions in the jaws.

The use of filters (algorithms) to refining the image is critical for sensitivity and specificity involved in the diagnosis of lytic areas of the jaws. The evaluation of the presence of bone destruction is very important, since it directs the prognosis and the planning and conducting of therapeutic process. High-resolution iCAT CBCT images resulted in an increase in sensitivity without jeopardizing specificity [19].

**Figure 2:**
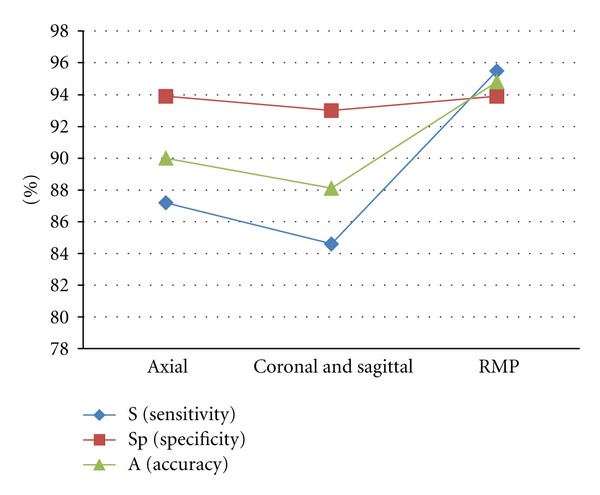
Evaluation of the sensitivity (S), specificity (Sp), and accuracy (A), according to the type of protocol, in the study of the presence or absence of simulated lesions.

Observation protocols play an important role in the analysis of mandibular bone lesions. The junction of viewing plans (MPR), in our study, shows excellent values of sensitivity, specificity, and accuracy, with respect to the assertion of the presence of simulated osteolytic lesions (Table 3 and Figure 2).

Analyses were done by regions of the mandible (buccal, lingual, and base), and with regard to verification of the presence of bone lesions, the values of the measures were correspondingly lower for lesions located in the lingual and higher for lesions found on the buccal (Table 4).

**Figure 3:**
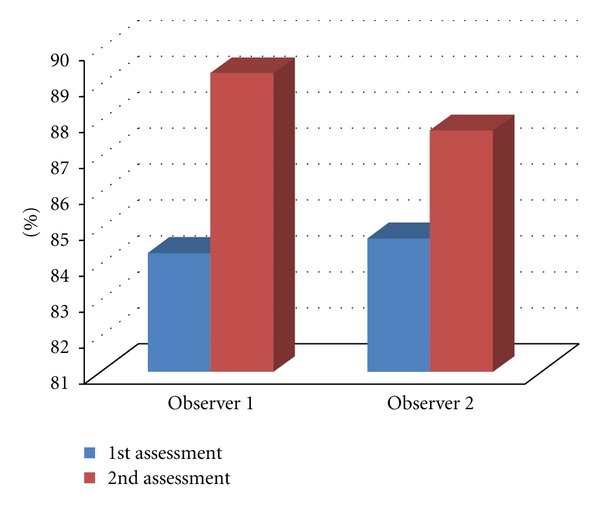
Agreement between the gold standard with each examiner and assessment, in the study of loci numbers.

In the present study, stands out the agreement between the examiners, in each assessment, with the gold standard, which ranged from 84.3% to 89.3%, whereas the agreement of each examiner was higher at the second moment of your assessment. Kappa values ranged from 0.77 (good agreement) to 0.84 (excellent agreement) (Table 5 and Figure 3).

In the evaluation of intraobserver agreement in the assessment of loci number, Table 6 shows that the percentage of agreement ranged from 84.9% to 89.9%, and kappa value being 0.85 (excellent agreement) in the evaluations of observer 1 and 0.77 (Good agreement) in the evaluations of observer 2.

In the evaluation of interobserver agreement (Table 7) compared to the assessment of loci number, it is possible to check that it was 96.2% in the first assessment and 96.4% in the second evaluation, with kappa values ranging from 0.94 to 0.95 (excellent agreement).

The analysis of intra- and interobservers kappa values showed excellence in most of the results, demonstrating the reproducibility of the method used. Thus, this was an *in vitro* study, and based on the values found, it can be a basis for further studies, including those performed *in vivo*.

## 5. Conclusions

- The sensitivity and specificity (validity) of cone beam computed tomography (CBCT) for diagnosis of osteolytic lesions (simulated) in the mandible, using standalone workstation, was shown.
- The influence of filters (algorithms) to improve the CBCT image was confirmed, and the very sharp filter was higher, which justifies its use for clinical activities, followed by normal and hard filters, in order to decrease values.
- The best protocol for visualization the simulated osteolytic lesions was the MPR.

## Figures and Tables

**Table 1:** Evaluation of the sensitivity (S), specificity (Sp), positive predictive value (PPV), negative predictive value (NPV), and accuracy (A) for the total group in the study of the presence or absence of simulated lesions.

S		Sp		PPV		NPV		A	
*N*	%	*N*	%	*N*	%	*N*	%	*N*	%
417/468	89.1	320/342	93.6	417/439	95.0	320/371	86.3	737/810	91.0

**Table 2:** Evaluation of the sensitivity (S), specificity (Sp), positive predictive value (PPV), negative predictive value (NPV), and accuracy (A), according to the type of filter, in the study of the presence or absence of simulated lesions.

Type of filter	S		Sp		PPV		NPV		A	
	*N*	%	*N*	%	*N*	%	*N*	%	*N*	%
Hard	135/156	86.5	105/114	92.1	135/144	93.8	105/126	92.1	240/270	88.9
Normal	134/156	85.9	103/114	90.4	134/145	92.4	103/125	82.4	237/270	87.8
Very sharp	148/156	94.9	112/114	98.2	148/150	98.7	112/120	93.3	260/270	96.3

**Table 3:** Evaluation of the sensitivity (S), specificity (Sp), positive predictive value (PPV), negative predictive value (NPV), and accuracy (A), according to the protocol type, in the study of the presence or absence of simulated lesions.

Protocol type	S		Sp		PPV		NPV		A	
	*N*	%	*N*	%	*N*	%	*N*	%	*N*	%
Axial	136/156	87.2	107/114	93.9	136/143	95.1	107/127	84.3	243/270	90.0
Coronal and sagital	132/156	84.6	106/114	93.0	132/140	94.3	106/130	81.5	238/270	88.1
MPR	149/156	95.5	107/114	93.9	149/156	95.5	107/114	93.9	256/270	94.8

**Table 4:** Evaluation of the sensitivity (S), specificity (Sp), positive predictive value (PPV), negative predictive value (NPV), and accuracy (A), according to theplace searched, in the study of the presence or absence of simulated lesions.

Place searched	S		Sp		PPV		NPV		A	
	*N*	%	*N*	%	*N*	%	*N*	%	*N*	%
Buccal	157/171	91.8	98/99	99.0	157/158	99.4	98/112	97.5	255/270	94.4
Lingual	135/153	88.2	100/117	85.5	135/152	88.8	100/118	84.7	235/270	87.0
Base	125/144	86.8	122/126	96.8	125/129	96.9	122/141	86.5	247/270	91.5

**Table 5:** Evaluation of agreement between the gold standard with each observer and assessment, in the study of loci numbers.

Observer	Assessment	Agreement		Kappa
		*n*/810	%	(Agreement rate of 95%)
1	1st	683	84.3	0.77 (0.73 to 0.81)
1	2nd	723	89.3	0.84 (0.81 to 0.87)
2	1st	686	84.7	0.77 (0.73 to 0.81)
2	2nd	710	87.7	0.81 (0.79 to 0.85)

**Table 6:** Evaluation of intraobserver agreement in the study of loci numbers.

Observer	Agreement		Kappa
	*n*/810	%	(AR of 95%)
1	728	89.9	0.85 (0.82 to 0.88)
2	688	84.9	0.77 (0.73 to 0.81)

**Table 7:** Evaluation of agreement between observers (interobserver) for each evaluation, in the study of loci numbers.

Assessment	Agreement		Kappa
	*n*/810	%	(AR of 95%)
1	779	96.2	0.94 (0.92 to 0.96)
2	781	96.4	0.95 (0.93 to 0.97)
